# Evaluation of eight kinds of flavor enhancer of umami taste by an electronic tongue

**DOI:** 10.1002/fsn3.2178

**Published:** 2021-02-18

**Authors:** Kai Wang, Haining Zhuang, Fangling Bing, Da Chen, Tao Feng, Zhimin Xu

**Affiliations:** ^1^ Technology Centre of China Tobacco Yunnan Industrial Co., Ltd. Kunming China; ^2^ Key Laboratory of Edible Fungi Resources and Utilization (South) Institute of Edible Fungi Shanghai Academy of Agricultural Sciences Ministry of Agriculture National Engineering Research Center of Edible Fungi Shanghai China; ^3^ School of Perfume and Aroma Technology Shanghai Institute of Technology Shanghai China; ^4^ Department of Food Science and Technology Ohio State University Columbus OH USA; ^5^ School of Nutrition and Food Sciences Louisiana State University Agricultural Center Baton Rouge LA USA

**Keywords:** discriminant factor analysis, electronic tongue, flavor enhancer, principal component analysis, sensory evaluation

## Abstract

The umami intensity of single flavor enhancer was detected by an electronic tongue and human sensory. The linear fit was performed to unravel the concentration–response values correlations of eight flavor enhancers. The electronic tongue response data were then analyzed using principal component analysis (PCA) and discriminant factor analysis (DFA) method. It was found that the umami response value of the electronic tongue and the flavor enhancer concentration showed a semi‐logarithmic function. Moreover, the PCA and DFA could successfully distinguish the variety and concentration of flavor enhancer. The umami intensities were also assessed by human sensory and showed consistency with those of electronic tongue tests. This implies the electronic tongue has a great potential as an alternative for biological tongue on sensing intensity of flavor enhancer.

## Graphical abstract

The umami response intensity of the electronic tongue was positively correlated with the concentration of all the umami substances and showed on a semi‐logarithmic function. The eight different flavor enhancers were successfully discriminated by PCA and DFA methods

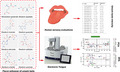

## 1 INTRODUCTION

Taste of foodstuff is a decisive factor for purchasing and consumers' acceptance of the food. Umami is among the most important tastes such as sourness, sweetness, bitterness, and saltiness (Stone & Herbert, 2012; Yamaguchi & Ninomiya, 2000). Umami substances are found naturally in various foods, including sea food (e.g., fish, seaweed, clam, and oyster), cheese, and vegetables (e.g., edible fungi, soybean, and carrot) (Shah et al., 2010). They could also be used as additives, with a well‐known commercial name‐flavor enhancer. The traditional flavor enhancers can be categorized into three main groups: amino acids and their sodium salts, nucleotides and their sodium salts, organic acids and their sodium salts. Of which, monosodium glutamate has been widely used for many years (Flavor Enhancers & Potentiators, 2006). It enables to enhance persistence, richness, thickness, and mouthfulness of foodstuff (Mccabe & Rolls, 2010; Yamaguchi, 1979) without introducing its own taste. Besides monosodium glutamate, sodium aspartate, disodium inosinate (IMP), disodium guanylate (GMP), disodium cytidylate (CMP), disodium adenylate (AMP), disodium uridylate (UMP), and disodium succinate are considered as flavor enhancers for food applications.

The quality of foods has been evaluated using many different analytical tools to identify their physical, chemical, and sensory characteristics. Sensory evaluation by panelists is among the most commonly used methods, which can reflect the overall acceptance of food products (Stone & Herbert, 2012). When eating and tasting, the stimulation of a substance to human tongue varies with its concentrations. A taste curve can be established to unreal the correlation between the concentration of the substance and the intensity of the taste (Tian et al., 2015). However, sensory evaluation was affected by various external factors and physical and psychological conditions of the panelist, which requires large number of participants in order to acquire meaningful data (Yang et al., 2013). Therefore, an objective and rapid taste evaluation method is of vital importance for industries who intend to quickly narrow down the range of flavor enhancer content in food products.

In recent years, the electronic tongue has been considered as an valuable tool to evaluate the food stuffs in industries to discriminate and quantify the compounds of the basic tastes (Yang et al., 2013). The electronic tongue tested the samples by simulating the human tongue concept, and meanwhile, it has higher sensitivity, better repeatability, and shorter determination time compared to the human tongue (Vlasov et al., 2002). Because of these advantages, the electronic tongue was applied in mineral waters (Labrador et al., 2009), tea (Chen et al., 2008), honey (Major et al., 2011), wines (Giorgio et al., 2007), beverages (Peres et al., 2009), and pharmaceuticals for the quality analysis and taste analysis. The electronic tongue enables to distinguish the intensity of bitterness and detect the taste‐masking effect (Zheng & Keeney, 2006). For evaluating umami taste, it was mainly used to rank the umami intensity of monosodium glutamate, disodium inosinate, and guanylate (Yang et al., 2013) and in quantification of umami in tomato (Katrien et al., 2008).

In this study, the relationship between the concentration and intensity of a single umami taste on the electronic tongue was examined. The potential of using the electronic tongue to compare and evaluate the different flavor enhancers was also investigated. To prove the accuracy and reliability of the electronic tongue, human sensory evaluation was conducted concomitantly. From the current study, we found the electronic tongue has great potential as a replacement for some human sensory evaluation without comprising the sensory results.

## 2 MATERIALS AND METHODS

### 2.1 Reagents and materials

The eight different commercial umami substances (food grade) including monosodium glutamate, sodium aspartate, disodium inosinate, disodium guanylate, disodiumcytidylate, disodium adenylate, disodium uridylate, and disodium succinate (food grade) were purchased from Tianfeng Food Technology Co., Ltd. in a form of white crystalline powder. Reagents of analytical grade, such as NaCl (0.1 mol/L), HCl (0.1 mol/L), and sodium L‐glutamate (0.1 mol/L) solution, were purchased from Alpha M. O. S. Inc. They were diluted to 0.01 mol/L with water before use (Giorgio et al., 2007).

### 2.2 Preparation of umami substance samples

Eight flavor enhancers were weighted into water to a certain concentration. For each flavor enhancer, a serious of concentrations were prepared (Table 1).

**TABLE 1:** The different concentrations of eight flavor enhancer samples

No.	Flavor enhancer	Concentration gradient (g/L)						
1	Monosodium glutamate (MSG)	0.12	0.17	0.24	0.34	0.49	0.70	1.00
2	Sodium aspartate	0.20	0.25	0.30	0.35	0.40	0.45	0.50
3	Disodium inosinate (IMP)	0.25	0.30	0.35	0.40	0.45	0.50	0.55
4	Disodium guanylate (GMP)	0.15	0.20	0.25	0.30	0.35	0.40	0.45
5	Disodium adenylate (AMP)	0.15	0.20	0.25	0.30	0.35	0.40	0.45
6	Disodium cytidylate (CMP)	0.50	0.55	0.60	0.65	0.70	0.75	0.80
7	Disodium uridylate (UMP)	0.45	0.50	0.55	0.60	0.65	0.70	0.75
8	Disodium succinate	0.30	0.35	0.40	0.45	0.50	0.55	0.60

### 2.3 Electronic tongue measurements

All samples were measured using the ASTREE electronic tongue (Alpha M. O. S., Toulouse, France) multisensory system equipped with an advanced chemometrics software package, and a 48‐position auto‐sampler (Metrohm, Ltd.). The electronic tongue used in this work comprised of seven chemical sensors. The taste sensor set consisted of UMS, SRS, SWS, STS, BRS, SPS, and GPS sensors to detect five basic tastes (umami, sourness, sweetness, saltiness, and bitterness) using an Ag/AgCl reference electrode (Metrohm, Ltd.) (Kang et al., 2014).

To achieve the best performance of the electronic tongue, the sensors were conditioned by a conditioning, calibration, and diagnostic process before analyzing each sample. 0.01 mol/L of sodium chloride (NaCl), sodium L‐glutamate (MSG), and hydrochloric acid (HCl) were used for the conditioning, diagnosis, and calibration processes (Yang et al., 2013). The E‐tongue detecting conditions were set the same as the sensory evaluation method. Distilled water was used for cleaning during the testing. The conditioning test was used for regenerating the activity of sensor coating. The calibration was applied to standardize the sensor values in each analysis and ensure the results of all the samples were consistently and comparably (Campos et al., 2012; Ciosek & Wróblewski, 2007). A diagnostic process was performed to measure the sensitivity and discriminating capability of the sensor.

All samples were analyzed at ambient temperature with a 10 s measurement time (20 ml volume) and 120 s rinse time for the reference electrode and seven sensors. A washable cycle was performed to clean the sensors before the next analysis (Tian et al., 2013). Each sample was measured six times by the sensors, and three stable equilibrium data points were recorded. All the experiments were completed continuously to avoid inconsistency caused by the aging degradation of sensors. Data acquisition and analysis were operated by software Astree II (Alpha M. O. S. V12.0).

### 2.4 Sensory evaluation

The flavor enhancers of various concentrations were prepared as described previously. Notably, only single flavor enhancer, rather than their mixtures were tested to reduce the difficulty of human sensory evaluation. In this way, the panelists can easily distinguish the concentrations of single flavor enhancer and score their intensity.

The eight flavor enhancers are all food grade, which are safe to panelists. Ethics involving human experiments has been approved by the ethic committee of Shanghai Institute of Technology. Twenty panelists recruited for this study were aged between 18 and 30 with good health, that is, no hypoglycaemia, diabetes, dentures, chronic colds, or sinusitis. The panelists were trained over a month to evaluate umami taste from flavor enhancers (Civille & Oftedal, 2012). Each panelist was isolated by an individual sensory evaluation booth and given 0.08, 0.34, and 1.00 g/L of MSG solution for training to perceive the intensity of umami taste, where the three concentrations represent low, medium, and high umami intensity, respectively, according to ISO3972:1991. Data from panelists who could not distinguish the umami intensity of the flavor were excluded. After training, ten individuals who met the health requirements were selected for sensory evaluation.

The sensory evaluation of the samples was performed in triplicates on different days. Eight samples were served in each session, and panelists were given a break between each session. In order to avoid temperature differences, all samples were kept and served at 45℃. Presentation order of the samples was randomized and balanced. Between the samples, panelist drank purified water until the taste was vanished.

The scale of the intensity was from 1 to 10, where 1 represents no umami intensity and 10 represents the highest umami intensity. The values given by ten panelists for the umami intensity were used for multivariate statistical analysis.

### 2.5 Statistics and data analysis

The data obtained from the electronic tongue were analyzed by PCA and DFA using the *α*‐Astree software. The PCA produces a score plot to visualize the differences among experiments and identify the main variables called components (Berrueta et al., 2007). The principal component (C1) and the second principal component (C2) were chosen in the present work. DFA was used to estimate the possibility of separating different groups (Winquist, 2008). The data used for the curve were calculated using a Macro Arithmetic Processor (commercial information). The response values were transformed into the value from 0 to 12.

The sensory data were calculated and statistically tested using the statistical analysis system (SAS 8.2) software (SAS Institute Inc.).

## 3 RESULTS AND DISCUSSION

### 3.1 Response pattern of electronic tongue to flavor enhancer of various concentrations

**FIGURE 1:**
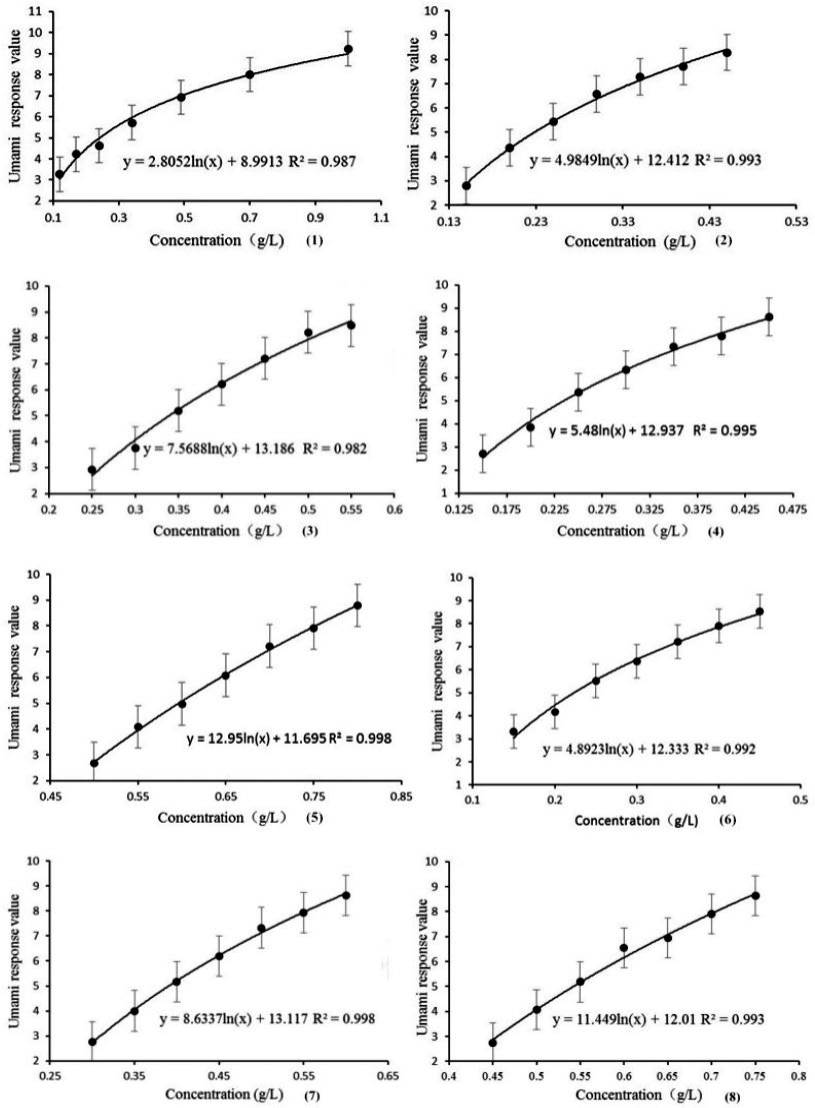
Changes of umami response values with the concentrations of the flavor enhancers. The correlation was fitted using a semi‐logarithmic function

The determination results for single taste of umami using the electronic tongue are shown in Figure 1. It can be seen that the umami response value increased with the increasing concentration of the flavor substances. A semi‐logarithmic function showed great fit for the umami response value–flavor substance concentration correlation. Similar observation was found previously on the sour and salty basic tastes (Tian et al., 2015). The coefficients of semi‐logarithmic functions were different for different flavor substances even though they had similar curve shape. Disodium adenylate had the highest coefficient, followed by disodium succinate and disodium uridylate. Monosodium glutamate had the lowest coefficient. These coefficients reflected the sensitivity of eight flavor enhancers toward the metal oxide membrane of sensors of electronic tongue. Neither molecular weight nor their chemical/physical properties (Table 2) showed direct correlation with the sensitivity. Most likely the interaction between the flavor substance and the metal oxide membranes is the decisive factor, which requires further study. In a previous study (Keast & Breslin, 2003), the tastes of sour, sweet, bitter, and salty were investigated at low, medium, and high concentrations. In here, a series of concentrations were used for each sample prior to modeling. The models could be used as a reference of evaluating the umami intensity in food. The result was consistent with the research of Tian et al. about five basic tastes.

**TABLE 2:** The sample concentration setting and its molecular properties of eight flavor enhancer samples

No.	Flavor enhancer	Concentration gradient (g/L)		Molecular structure	M_W_	HBDC	HBAC	RBC	TPSA ( Å^2^)
1	Monosodium glutamate (MSG)	0.5	1.0	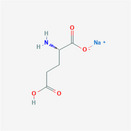	169.11	2	5	4	103
2	Disodium aspartate	0.5	1.0	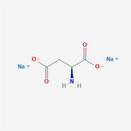	177.07	1	5	1	106
3	disodium inosinate (IMP)	0.5	1.0	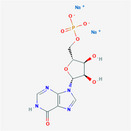	392.17	3	10	3	181
4	Disodium guanylate	0.5	1.0	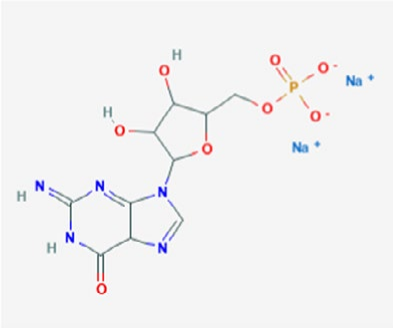	425.2	5	11	3	204
5	Disodium adenylate (AMP)	0.5	1.0	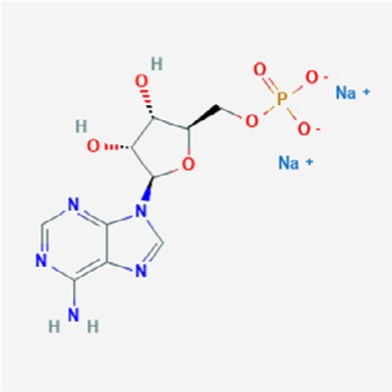	391.19	3	11	3	192
6	Disodium cytidylate	0.5	1.0	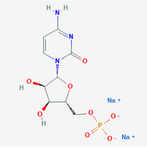	367.16	3	8	3	181
7	Disodium uridylate (UMP)	0.5	1.0	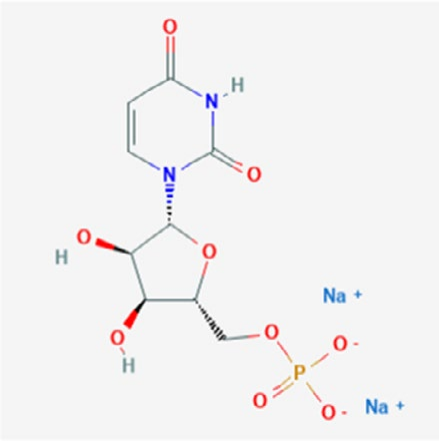	368.14	3	9	3	172
8	Disodium succinate	0.5	1.0	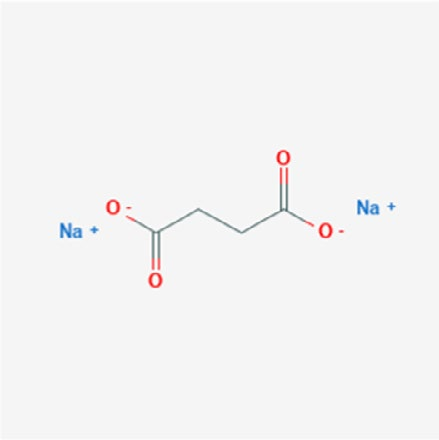	162.05	0	4	1	80.3

Abbreviations: HBAC, Hydrogen Bond Acceptor Count; HBDC, Hydrogen Bond Donor Count; RBC, Rotatable Bond Count; TPSA, Topological Polar Surface Area.

### 3.2 Electronic tongue analysis of the flavor enhancer of same concentration gradients

**FIGURE 2:**
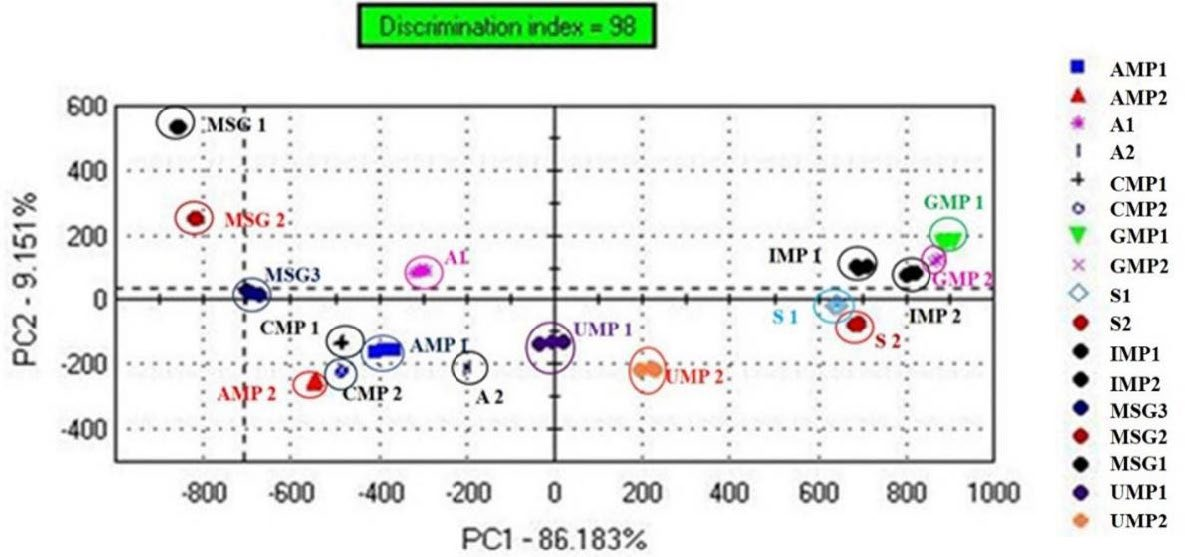
Principal component analysis (PCA) score plot of the eight flavor enhancer solutions. The samples are the same as those in Table 2. AMP1, 0.5 g/L disodium adenylate; AMP2, 1.0 g/L disodium adenylate; A1, 0.5 g/L sodium aspartate; A2, 1.0 g/L sodium aspartate; CMP1, 0.5 g/L disodium cytidylate; CMP2, 1.0 g/L disodium cytidylate; GMP1, 0.5 g/L disodium guanylate; GMP2, 1.0 g/L disodium guanylate; S1, 0.5 g/L disodium succinate; S2, 1.0 g/L disodium succinate; IMP1, 0.5 g/L disodium inosinate; IMP2, 1.0 g/L disodium inosinate; MSG1, 0.5 g/L monosodium glutamate; MSG2, 1.0 g/L monosodium glutamate; MSG3, 2.0 g/L monosodium glutamate; UMP1, 0.5 g/L disodium uridylate; UMP2, 1.0 g/L disodium uridylate

The discriminative ability of electronic tongue system in distinguishing the umami taste of eight different flavor enhancers was examined. The PCA was performed on the raw data from the electronic tongue, and the principal component score vectors were extracted as the input of the pattern recognition. Therefore, it can extract the useful information by elimination of overlapped data. The responses of the sensor array were used to create a matrix with 51 rows (17 samples × 3 batches) and seven columns (7 sensor outputs). The PCA was conducted on the matrix, and the results were shown in Figure 2. The relative contributions of the PC1 and PC2 were 86.18% and 9.15%, respectively. The cumulative contribution percentage of the two was 95.33%, which enables to provide most of the information. There was a clear discrimination among all the samples with a discrimination index (DI) of 98. All the samples had a lower dispersion with a higher distinction. Samples from the same flavor substance were closely located in the PCA plot, whereas different substances were distributed far away from each other. For the same flavor substance, the samples distributed along C2 axis in an order of low concentration on the top of high concentrations. These findings indicate the high sensitivity of electronic tongue on discrimination of not only different flavor substances but also same flavor substance with different concentrations.

**FIGURE 3:**
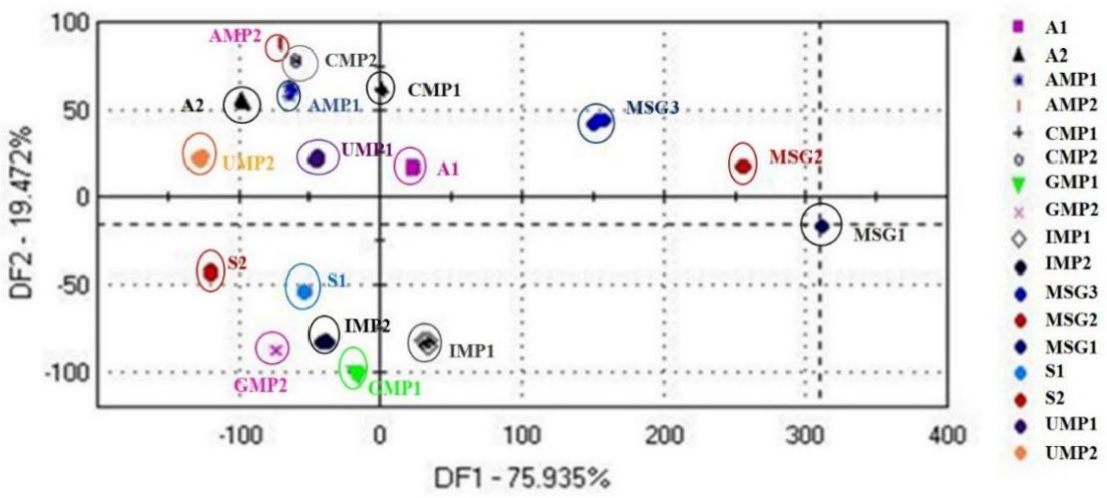
Discriminant factor analysis (DFA) score plot of the eight flavor enhancer solutions. The samples are the same as those in Table 2. A1, 0.5 g/L sodium aspartate; A2, 1.0 g/L sodium aspartate; AMP1, 0.5 g/L disodium adenylate; AMP2, 1.0 g/L disodium adenylate; CMP1, 0.5 g/L disodium cytidylate; CMP2,1.0 g/L disodiumcytidylate; GMP1, 0.5 g/L disodium guanylate; GMP2, 1.0 g/L disodium guanylate; IMP1, 0.5 g/L disodium inosinate; IMP2,1.0 g/L disodium inosinate; MSG1, 0.5 g/L monosodium glutamate; MSG2, 1.0 g/L monosodium glutamate; MSG3, 2.0 g/L monosodium glutamate; S1, 0.5 g/L disodium succinate; S2, 1.0 g/L disodium succinate; UMP1, 0.5 g/Ldisodium uridylate; UMP2, 1.0 g/Ldisodium uridylate

The DFA was also performed on the taste sensor data from the electronic tongue (Figure 3). The cumulative contribution percentage of two discriminant factors was 95.41%. It was found that the distribution maps of the samples from the same flavor substance were much smaller than those of PCA. But for different flavor substances, DFA had better distinguishable capacity better than those of PCA. Our finding agrees well with that of Wei (Wei et al., 2013) who also analyzed by principal component analysis (PCA) and discriminant function analysis (DFA) for category classification.

### 3.3 Sensory evaluation of the umami intensity of flavor enhancers

The umami intensity of each sample obtained by the electronic tongue analysis was also assessed by sensory, and the result was shown in Table 3. The umami score of the flavor substances from the human sensory evaluation ranged from 1.5 (sodium aspartate) to 8.6 (disodium adenylate). The umami score of the flavor substance at 1.0 g/L was higher than those at 0.5 g/L. No significant difference was found on monosodium glutamate, sodium aspartate, and disodium uridylate regarding the umami score. The umami intensity (No.1‐No.8) from the E‐tongue sensory test was in a range from 2.24 (No.2, 0.5 g/L sodium aspartate) to 7.2 (No.5, 0.5 g/L disodium adenylate), while the umami intensity (No.9‐No.16) was in a range from 5.79 (No.15, 1.0 g/L disodium uridylate) to 9.21 (No.13, 1.0 g/L disodium adenylate). The umami intensity of disodium adenylate (No.5, 0.5 g/L disodium adenylate and No.15, 1.0 g/L disodium uridylate) was higher than the other flavor enhancers. The samples of (No.1, 0.5 g/L monosodium glutamate and No.7, 0.5 g/L disodium uridylate) have no significant difference in umami intensity (*p* < .05). The higher umami taste score has the higher E‐tongue sensory value. The result suggested that human sensory evaluation and electronic tongue detection were consistent in the evaluation of the umami taste intensity.

**TABLE 3:** Umami taste intensity based on electronic tongue test and human sensory evaluations

No.	Samples	Human sensory test	E‐tongue sensory test
1	Monosodium glutamate (0.5 g/L)	2.7 ± 1.16 gf	4.22 ± 0.44 j
2	Sodium aspartate (0.5 g/L)	1.5 ± 0.71 g	2.24 ± 0.81 h
3	Disodium inosinate (0.5 g/L)	5.6 ± 1.84 e	6.24 ± 0.54 i
4	Disodium guanylate (0.5 g/L)	6.1 ± 1.66 e	6.76 ± 0.65 f
5	Disodium adenylate (0.5 g/L)	6.4 ± 1.71 cde	7.20 ± 0.34 e
6	Disodium cytidylate (0.5 g/L)	3.2 ± 1.23 f	5.08 ± 0.24 k
7	Disodium uridylate (0.5 g/L)	2.5 ± 1.27 gf	4.09 ± 0.23 j
8	Disodium succinate (0.5 g/L)	5.8 ± 1.87 de	6.44 ± 0.42 i
9	Monosodium glutamate (1.0 g/L)	7.1 ± 1.45 bcd	7.91 ± 0.76 d
10	Sodium aspartate (1.0 g/L)	5.2 ± 1.69 e	6.19 ± 0.50 i
11	Disodium inosinate (1.0 g/L)	7.3 ± 1.49 abc	8.15 ± 1.01 c
12	Disodium guanylate (1.0 g/L)	7.2 ± 1.75 abcd	7.95 + 1.22 d
13	Disodium adenylate (1.0 g/L)	8.6 ± 0.97 a	9.21 ± 0.51 a
14	Disodium cytidylate (1.0 g/L)	6.4 ± 1.65 cde	6.54 ± 0.13 i
15	Disodium uridylate (1.0 g/L)	3.6 ± 1.17 f	5.79 ± 0.17 g
16	Disodium succinate (1.0 g/L)	7.9 ± 1.20 ab	8.56 ± 0.30 b

Note

Value is expressed as mean + standard deviation of triplicate analysis; means with the same letter are not significantly different between themselves (*p* < .05) according to Duncan's multiple range test.

## 4 CONCLUSION

In this study, the electronic tongue analysis was able to identify the eight different flavor enhancers. Their umami response intensity from electronic tongue was positively correlated with their concentration showing as a semi‐logarithmic function. The eight different flavor enhancers were successfully discriminated by PCA and DFA methods. The PCA was superior to DFA in discriminating the same flavor enhancer at different concentrations, while DFA method was more suitable to distinguish different types of flavor enhancers. Human sensory test further confirmed the accuracy of the electronic tongues on the analysis of the flavor enhancers. This indicates the electronic tongue is a valuable tool to rapidly analyze the flavor as a partial replacement for human sensory.

## CONFLICT OF INTEREST

The authors declare no conflict of interest.

## ETHICAL APPROVAL

This study does not involve any human or animal testing.

## Data Availability

Research data are not shared.

## References

[fsn32178-bib-0001] Berrueta, L. A. , Alonso‐Salces, R. M. , & Héberger, K. (2007). Supervised pattern recognition in food analysis. Journal of Chromatography A, 1158(1–2), 196–214. 10.1016/j.chroma.2007.05.024. 17540392

[fsn32178-bib-0002] Campos, I. , Alcañiz, M. , Aguado, D. , Barat, R. , Ferrer, J. , Gil, L. , Marrakchi, M. , Martínez‐Mañez, R. , Soto, J. , & Vivancos, J. L. (2012). A voltammetric electronic tongue as tool for water quality monitoring in wastewater treatment plants. Water Research, 46(8), 2605–2614. 10.1016/j.watres.2012.02.029. 22424964

[fsn32178-bib-0003] Chen, Q. , Zhao, J. , & Vittayapadung, S. (2008). Identification of the green tea grade level using electronic tongue and pattern recognition. Food Research International, 41(5), 500–504. 10.1016/j.foodres.2008.03.005.

[fsn32178-bib-0004] Ciosek, P. , & Wróblewski, W. (2007). Sensor arrays for liquid sensing–electronic tongue systems. Analyst, 132(10), 963–978. 10.1039/b705107g. 17893798

[fsn32178-bib-0005] Civille, G. V. , & Oftedal, K. N. (2012). Sensory evaluation techniques — Make "good for you" taste "good". Physiology & Behavior, 107(4), 598–605. 10.1016/j.physbeh.2012.04.015. 22554616

[fsn32178-bib-0006] Considine, D.M. , & Considine, G.D. (2006). Flavor enhancers and potentiators. In D.M. Considine & G.D. Considine (Eds.), Van Nostrand’s Scientific Encyclopedia, Food Technology 9th edn New York: John Wiley & Sons Inc. https://onlinelibrary.wiley.com/doi/abs/10.1002/0471743984.vse8048.

[fsn32178-bib-0007] Giorgio, V. , Larisa, L. , Roberto, P. , Corrado, D. N. , & Arnaldo, D. A. (2007). Metalloporphyrin ‐ based electronic tongue: An application for the analysis of Italian White wines. Sensors, 7(11), 2750–2762. 10.3390/s7112750. 28903259 PMC3965223

[fsn32178-bib-0008] Kang, B. S. , Lee, J. E. , & Park, H. J. (2014). Electronic tongue‐based discrimination of Korean rice wines (makgeolli) including prediction of sensory evaluation and instrumental measurements. Food Chemistry, 151, 317–323. 10.1016/j.foodchem.2013.11.084. 24423539

[fsn32178-bib-0009] Beullens, K. , Mészáros, P. , Vermeir, S. , Kirsanov, D. , Legin, A. , Buysens, S. , Cap, N. , Nicolaï, B. M. , & Lammertyn, J. (2008). Analysis of tomato taste using two types of electronic tongues. Sensors and Actuators B: Chemical, 131(1), 10–17. 10.1016/j.snb.2007.12.024.

[fsn32178-bib-0010] Keast, R. S. J. , & Breslin, P. A. S. (2003). An overview of taste–taste interactions. Food Quality & Preference, 14(2), 111–124.

[fsn32178-bib-0011] Labrador, R. , Soto, J. , Martínez‐Máez, R. , & Gil, L. (2009). An electronic tongue for qualitative and quantitative analyses of anions in natural waters. Journal of Applied Electrochemistry, 39(12), 2505. 10.1007/s10800-009-9942-y.

[fsn32178-bib-0012] Major, N. , Marković, K. , Krpan, M. , Šarić, G. , Hruškar, M. , & Vahčić, N. (2011). Rapid honey characterization and botanical classification by an electronic tongue. Talanta, 85(1), 569–574. 10.1016/j.talanta.2011.04.025. 21645743

[fsn32178-bib-0013] Mccabe, C. , & Rolls, E. T. (2010). Umami: A delicious flavor formed by convergence of taste and olfactory pathways in the human brain. European Journal of Neuroscience, 25(6), 1855–1864. 10.1111/j.1460-9568.2007.05445.x. 17432971

[fsn32178-bib-0014] Peres, A. M. , Dias, L. G. , Barcelos, T. P. , Sá Morais, J. , & Machado, A. (2009). An electronic tongue for juice level evaluation in non‐alcoholic beverages. Procedia Chemistry, 1(1), 1023–1026. 10.1016/j.proche.2009.07.255.

[fsn32178-bib-0015] Shah, A. K. M. A. , Ogasawara, M. , Egi, M. , Kurihara, H. , & Takahashi, K. (2010). Identification and sensory evaluation of flavour enhancers in Japanese traditional dried herring (Clupea pallasii) fillet. Food Chemistry, 122(1), 249–253. 10.1016/j.foodchem.2010.02.072.

[fsn32178-bib-0016] Stone, H. , Bleibaum, R.N. , & Thomas, H.A. (2012). H. Stone R.N. Bleibaum & H.A. Thomas Test Strategy and the Design of Experiments. Sensory Evaluation Practices, Food Science and Technology 4th edn. Test Strategy and the Design of Experiments, 117–165). Amsterdam: Elsevier. https://www.sciencedirect.com/science/article/pii/B9780123820860000042.

[fsn32178-bib-0017] Tian, H. , Feng, T. , Xiao, Z. , Song, S. , Li, Z. , Liu, Q. , Mao, D. , & Li, F. (2015). Comparison of intensities and binary interactions of four basic tastes between an electronic tongue and a human tongue. Food Science and Biotechnology, 24(5), 1711–1715. 10.1007/s10068-015-0222-9

[fsn32178-bib-0018] Tian, X. , Wang, J. , & Zhang, X. (2013). Discrimination of preserved licorice apricot using electronic tongue. Mathematical and Computer Modelling, 58(3–4), 743–751. 10.1016/j.mcm.2012.12.034.

[fsn32178-bib-0019] Vlasov, Y. , Legin, A. , & Rudnitskaya, A. (2002). Electronic tongues and their analytical application. Analytical & Bioanalytical Chemistry, 373(3), 136–146. 10.1007/s00216-002-1310-2. 12043015

[fsn32178-bib-0020] Wei, Z. , Wang, J. , & Jin, W. (2013). Evaluation of varieties of set yogurts and their physical properties using a voltammetric electronic tongue based on various potential waveforms. Sensors & Actuators B Chemical, 177, 684–694. 10.1016/j.snb.2012.11.056.

[fsn32178-bib-0021] Winquist, F. (2008). Voltammetric electronic tongues ‐ Basic principles and applications. Microchimica Acta, 163(1), 3–10. 10.1007/s00604-007-0929-2.

[fsn32178-bib-0022] Yamaguchi, S. (1979). Shizuko Y. The Umami Taste. Food Taste Chemistry, ACS Symposium Series 115, (33–51). American Chemical Society: Washington, D.C.. https://pubs.acs.org/doi/10.1021/bk‐1979‐0115.ch002.

[fsn32178-bib-0023] Yamaguchi, S. , & Ninomiya, K. (2000). Umami and palatability. Journal of Nutrition, 130(4S Suppl), 921S–926S. 10.1093/jn/130.4.921S 10736353

[fsn32178-bib-0024] Yang, Y. , Chen, Q. , Shen, C. , Zhang, S. , Gan, Z. , Hu, R. , Zhao, J. , & Ni, Y. (2013). Evaluation of monosodium glutamate, disodium inosinate and guanylate umami taste by an electronic tongue. Journal of Food Engineering, 116(3), 627–632. 10.1016/j.jfoodeng.2012.12.042.

[fsn32178-bib-0025] Zheng, J. Y. , & Keeney, M. P. (2006). Taste masking analysis in pharmaceutical formulation development using an electronic tongue. International Journal of Pharmaceutics, 310(1–2), 118–124. 10.1016/j.ijpharm.2005.11.046. 16431048

